# Supplementary material for: Tax1bp1 enhances bacterial virulence and promotes inflammatory responses during Mycobacterium tuberculosis infection of alveolar macrophages
Source: bioRxiv. 2024 Dec 16:2024.12.16.628616. Preprint. [Version 1] doi: 10.1101/2024.12.16.628616 (PMC11702572; doi:10.1101/2024.12.16.628616)
Supplement: Supplement 1 [file NIHPP2024.12.16.628616v1-supplement-1.pdf]

bioRxiv preprint doi: https://doi.org/10.1101/2024.12.16.628616; this version posted December 16, 2024. The copyright holder for this preprint (which was not certified by peer review) is the author/funder, who has granted bioRxiv a license to display the preprint in perpetuity. It is made available under aCC-BY 4.0 International license.

Supplemental Figure Legends

Figure 1-figure supplement 1. Tax1bp1 contributes to *M. tuberculosis* virulence and inflammatory cytokine responses. (A) In an independent experiment, male and female mice infected by the aerosol route with *M. tuberculosis* were euthanized at 1-, 9-, 21-, and 50 days post-infection. Lung homogenates were enumerated for CFU. Results are the mean ± SEM from five mice. The mean experimental inoculum was 104 CFU as determined by CFU enumeration at 1-day post-infection. (B) Cytokine levels from infected lung homogenates at 9-, 21-, and 50 days post-infection were measured by ELISA. Results are the mean ± SEM from five samples. The p-values from t-test comparisons are shown.

Figure 1-figure supplement 2. Lung pathology and neutrophil recruitment were similar during *M. tuberculosis* aerosol infection of wild-type and *Tax1bp1*$^{-/-}$ mice. (A) Paraffin-embedded thin sections of lung samples from infected wild-type and *Tax1bp1*$^{-/-}$ mice were stained with hematoxylin and eosin (H&E). (B) Pathology was analyzed in H&E-stained images from five infected wild-type and five *Tax1bp1*$^{-/-}$ mice at 21- and 50 days post-infection. (C) Paraffin-embedded thin sections of the lung from infected wild-type and *Tax1bp1*$^{-/-}$ mice were stained with antibodies against myeloperoxidase. Antibody staining was detected with 3,3'-diaminobenzidine. (D) Quantitative analysis of the percentage of cells that stained

bioRxiv preprint doi: https://doi.org/10.1101/2024.12.16.628616; this version posted December 16, 2024. The copyright holder for this preprint (which was not certified by peer review) is the author/funder, who has granted bioRxiv a license to display the preprint in perpetuity. It is made available under aCC-BY 4.0 International license.

positive for myeloperoxidase is shown. Results are the mean ± SEM from five mice. Brackets indicate p-values from t-test comparisons.

Figure 1-figure supplement 3. Ubiquitin colocalizes with *M. tuberculosis* in the lungs during murine aerosol infection of wild-type and *Tax1bp1*$^{-/-}$ mice. (A) Serial thin sections of paraffin-embedded lung specimens were stained with antibodies against ubiquitin, *M. tuberculosis*, or hematoxylin and eosin. Antibodies were detected with 3,3'-diaminobenzidine. (B) Quantitative analysis of ubiquitin staining pixel overlap with *M. tuberculosis* in overlayed images. Results are mean ± SEM from five samples. The p-value from the t-test comparison is shown.

Figure 2-figure supplement 1. Tax1bp1 enhances *L. monocytogenes* growth during murine infection. In an independent experiment, mice were infected with *L. monocytogenes* by the intravenous route, and CFU enumerated from spleen and liver homogenates at 48 hours post-infection. Results are the mean ± SEM from five mice. Brackets indicate p-values from t-test comparisons. CFU data were logarithmically transformed prior to statistical analysis.

Figure 2-figure supplement 2. Tax1bp1 promotes the formation of microabscesses and lymphocyte depletion during *L. monocytogenes* infection. (A, B) Serial thin sections of paraffin-embedded spleen and liver specimens from mice infected by the intraperitoneal route collected at 72 hours post-infection were stained with hematoxylin and eosin. (C-E) Pathology was analyzed in H&E-stained images from five infected wild-type and 5 *Tax1bp1*$^{-/-}$ mice at 72 hours post-infection.

Figure 3-figure supplement 1. Gating strategy used to identify myeloid subsets. A representative flow panel is shown depicting the gating strategy for identification and sorting of myeloid subsets. B, T, and NK cells were gated out. AMs (CD11b^{lo}CD11c^{hi}SiglecF^{hi}), MNC1 (SiglecF^{-}CD11b^{+}CD11c^{lo}MHCII^{+}), MNC2 (SiglecF^{-}CD11b^{+}CD11c^{hi}MHCII^{hi}), and neutrophils (Neut; SiglecF^{-}Ly6G^{hi}CD11b^{hi}) were sorted. Sorted cells were plated for *Mtb* CFU enumeration. The gating strategy used to identify ZsGreen-positive cells is shown in the bottom row.

Figure 3-figure supplement 2. Tax1bp1 promotes *Mtb* growth in AMs, PMNs, and MNC2 following low-dose aerosol infection. In an independent experiment, mice were infected with aerosolized *Mtb*

bioRxiv preprint doi: https://doi.org/10.1101/2024.12.16.628616; this version posted December 16, 2024. The copyright holder for this preprint (which was not certified by peer review) is the author/funder, who has granted bioRxiv a license to display the preprint in perpetuity. It is made available under aCC-BY 4.0 International license.

expressing ZsGreen. Five wild-type and five *Tax1bp1*$^{-/-}$ mice were euthanized at 1-, 7-, 14-, and 21 days post-infection. CFU were measured from lung homogenates at 1 day post-infection, which revealed the mean infectious dose of *Mtb* was 224 CFU/mouse. At 7-, 14-, and 21 days post-infection, lung cells were pooled and stained for AMs, PMNs, and recruited monocyte 1 and 2 subsets (MNC1, 2). The number of ZsGreen-positive counts from innate immune cells was quantified by analytical flow cytometry. Data were normalized to the number of cells analyzed.

Figure 6-figure supplement 1. Pathogen and host differential gene expression analysis volcano plots. Volcano plots display the differentially regulated genes from (A) *Mtb* and (B) the host during wild-type and *Tax1bp1*$^{-/-}$ AM infection with *Mtb*. The volcano plots display the $\log_2$fold change of normalized mean hit counts in wild-type vs. *Tax1bp1*$^{-/-}$ samples and $-\log_{10}$(adj. p-value for host genes or unadjusted p-value for *Mtb* genes). Colors denote genes that were upregulated (purple) or downregulated (green) in wild-type compared to *Tax1bp1*$^{-/-}$ samples.

Figure 8-figure supplement 1. Tax1bp1 enhances necrotic-like cell death and delays apoptosis during *Mtb* infection of AMs. As described in the Figure 8 legend, AMs were infected with *Mtb* at a M.O.I. of 1 in the presence of PI (propidium iodide) and CellEvent without (A) or with (B) IFN-γ added to the media. Fluorescence images were obtained at 20X magnification in two positions per well in three replicate wells. Representative fluorescence and brightfield microscopy images are displayed at days 1-8 post-infection. The white bar is 100 μm.

Figure 8-figure supplement 2. Tax1bp1 does not promote necrotic-like cell death during *Mtb* infection of BMDMs. BMDMs were infected with *Mtb* at a M.O.I. of 1 in the presence of PI (propidium iodide) and CellEvent without (A, C) or with (B, D) IFN-γ added to the media. Fluorescence images were obtained at 20X magnification in two positions per well in three replicate wells. Representative fluorescence and brightfield microscopy images are displayed at days 1-8 post-infection. The white bar denotes 100 μm. (C, D) The number of fluorescent cells in each field was quantified in the green (CellEvent) and red fluorescence (PI) channels. Mean, SEM, and statistically significant FDR-adjusted p-values comparisons are displayed. For clarity, only statistically significant p-values ($p \leq 0.05$) are displayed.

bioRxiv preprint doi: https://doi.org/10.1101/2024.12.16.628616; this version posted December 16, 2024. The copyright holder for this preprint (which was not certified by peer review) is the author/funder, who has granted bioRxiv a license to display the preprint in perpetuity. It is made available under aCC-BY 4.0 International license.

Figure 8-figure supplement 1. Model describing Tax1bp1's function during *Mtb* infection of AMs. Tax1bp1 enhances *Mtb* growth, inflammatory cytokine synthesis, PGE_2 production, and necrotic-like host cell death in AMs. Tax1bp1-deficiency, or expression of phosphosite-deficient Tax1bp1, decreases *Mtb* growth in AMs.

bioRxiv preprint doi: https://doi.org/10.1101/2024.12.16.628616; this version posted December 16, 2024. The copyright holder for this preprint (which was not certified by peer review) is the author/funder, who has granted bioRxiv a license to display the preprint in perpetuity. It is made available under aCC-BY 4.0 International license.

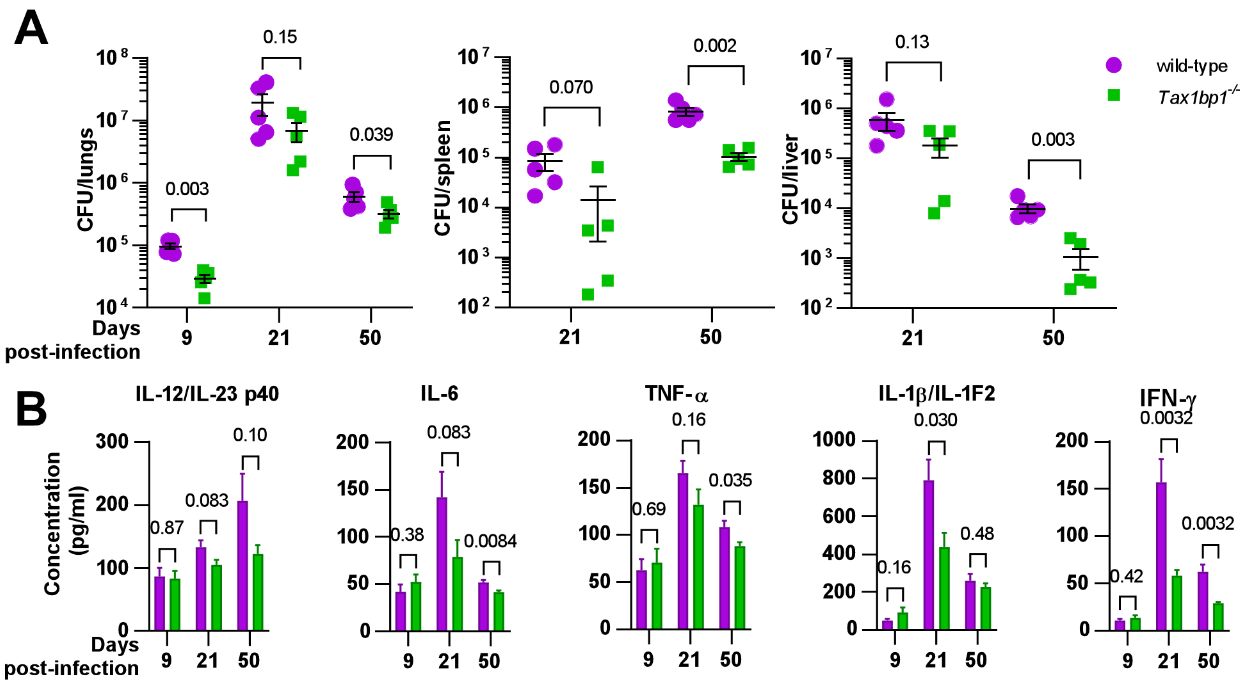

Figure 1-figure supplement 1

bioRxiv preprint doi: https://doi.org/10.1101/2024.12.16.628616; this version posted December 16, 2024. The copyright holder for this preprint (which was not certified by peer review) is the author/funder, who has granted bioRxiv a license to display the preprint in perpetuity. It is made available under aCC-BY 4.0 International license.

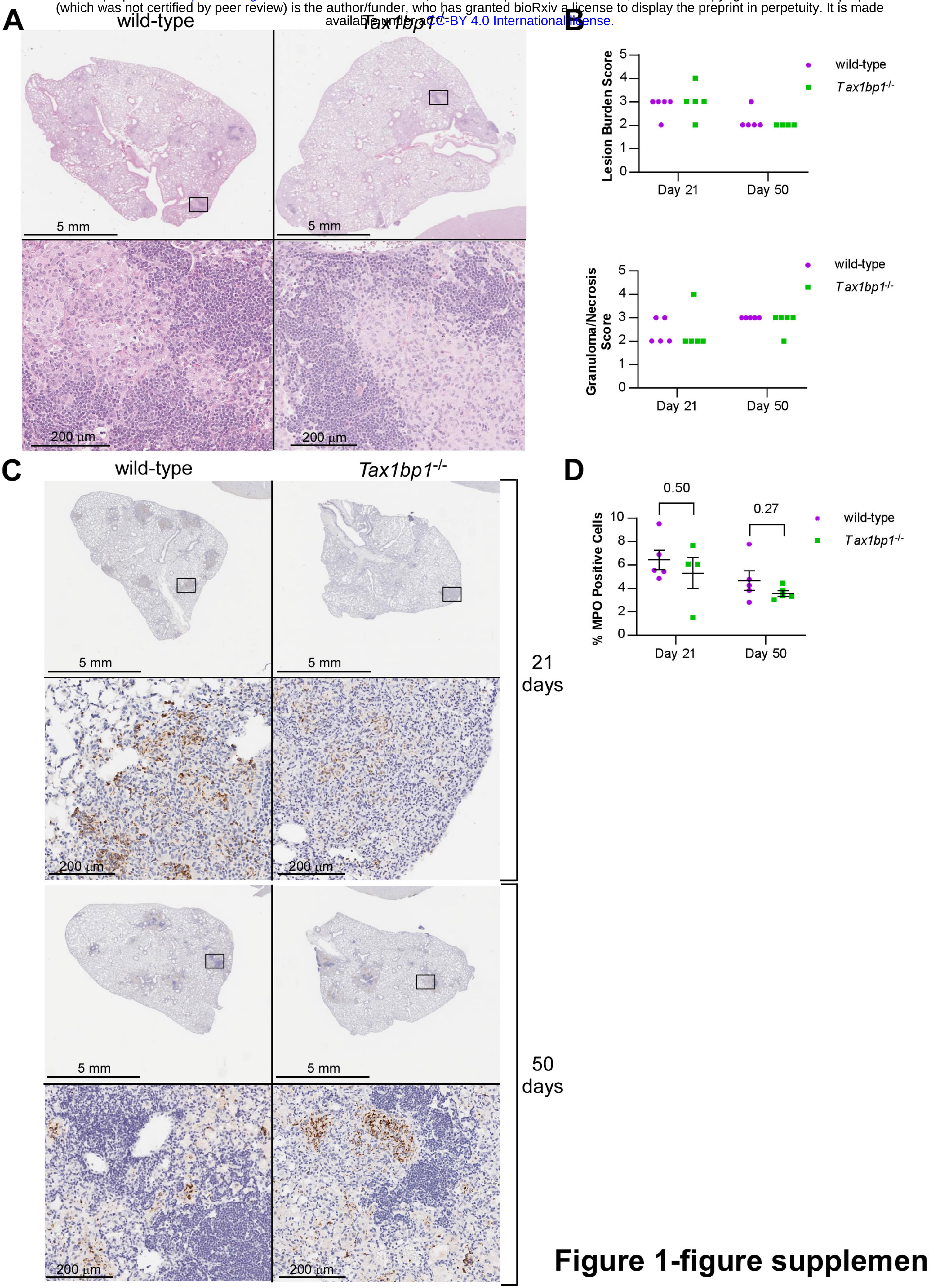

Figure 1-figure supplement 2

bioRxiv preprint doi: https://doi.org/10.1101/2024.12.16.628616; this version posted December 16, 2024. The copyright holder for this preprint (which was not certified by peer review) is the author/funder, who has granted bioRxiv a license to display the preprint in perpetuity. It is made available under aCC-BY 4.0 International license.

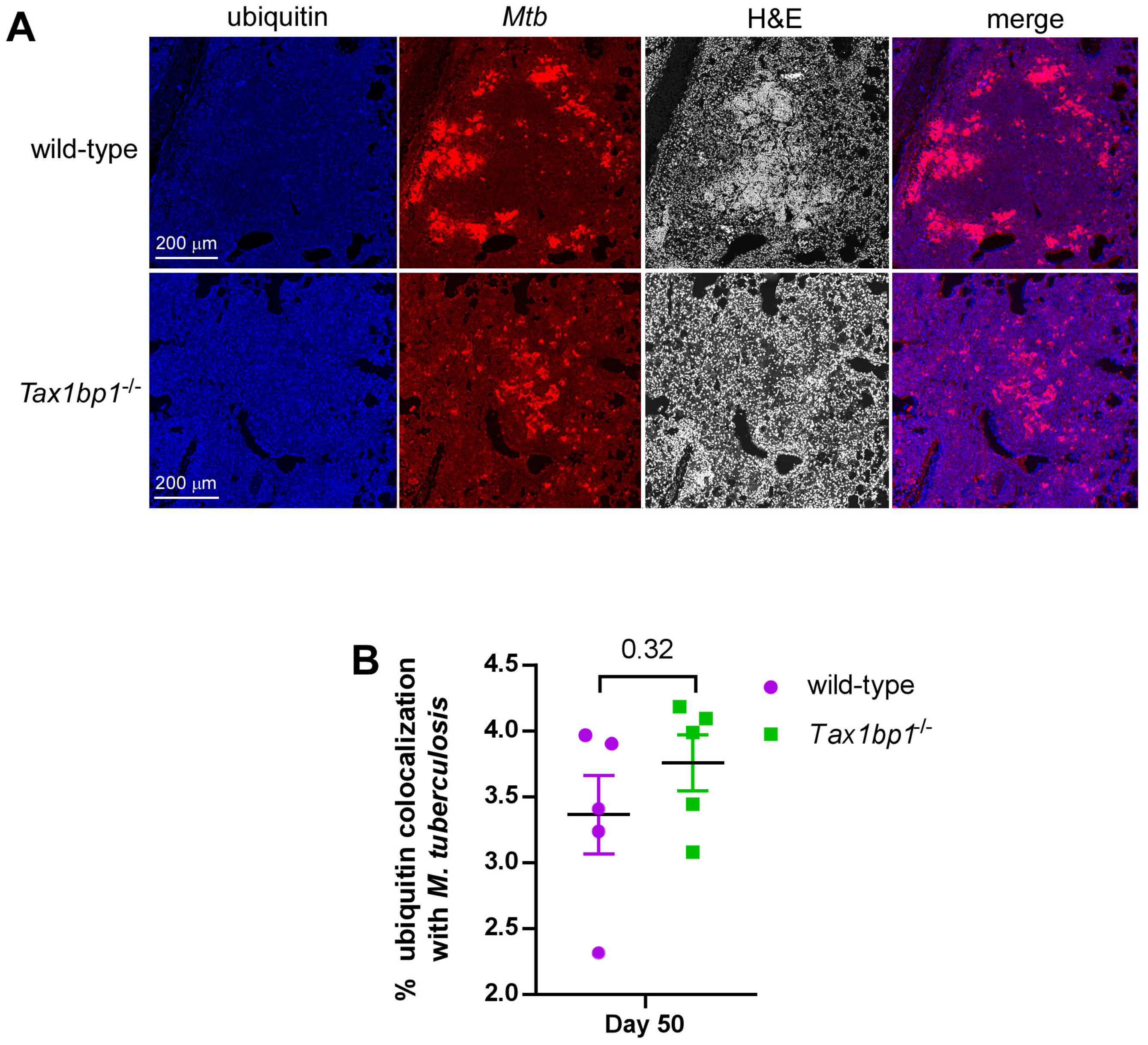

Figure 1-figure supplement 3

bioRxiv preprint doi: https://doi.org/10.1101/2024.12.16.628616; this version posted December 16, 2024. The copyright holder for this preprint (which was not certified by peer review) is the author/funder, who has granted bioRxiv a license to display the preprint in perpetuity. It is made available under aCC-BY 4.0 International license.

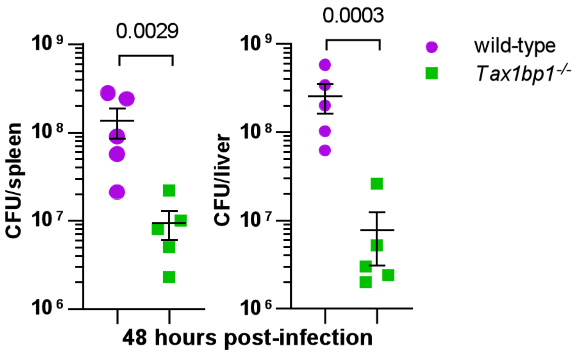

Figure 2-figure supplement 1

bioRxiv preprint doi: https://doi.org/10.1101/2024.12.16.628616; this version posted December 16, 2024. The copyright holder for this preprint (which was not certified by peer review) is the author/funder, who has granted bioRxiv a license to display the preprint in perpetuity. It is made available under aCC-BY 4.0 International license.

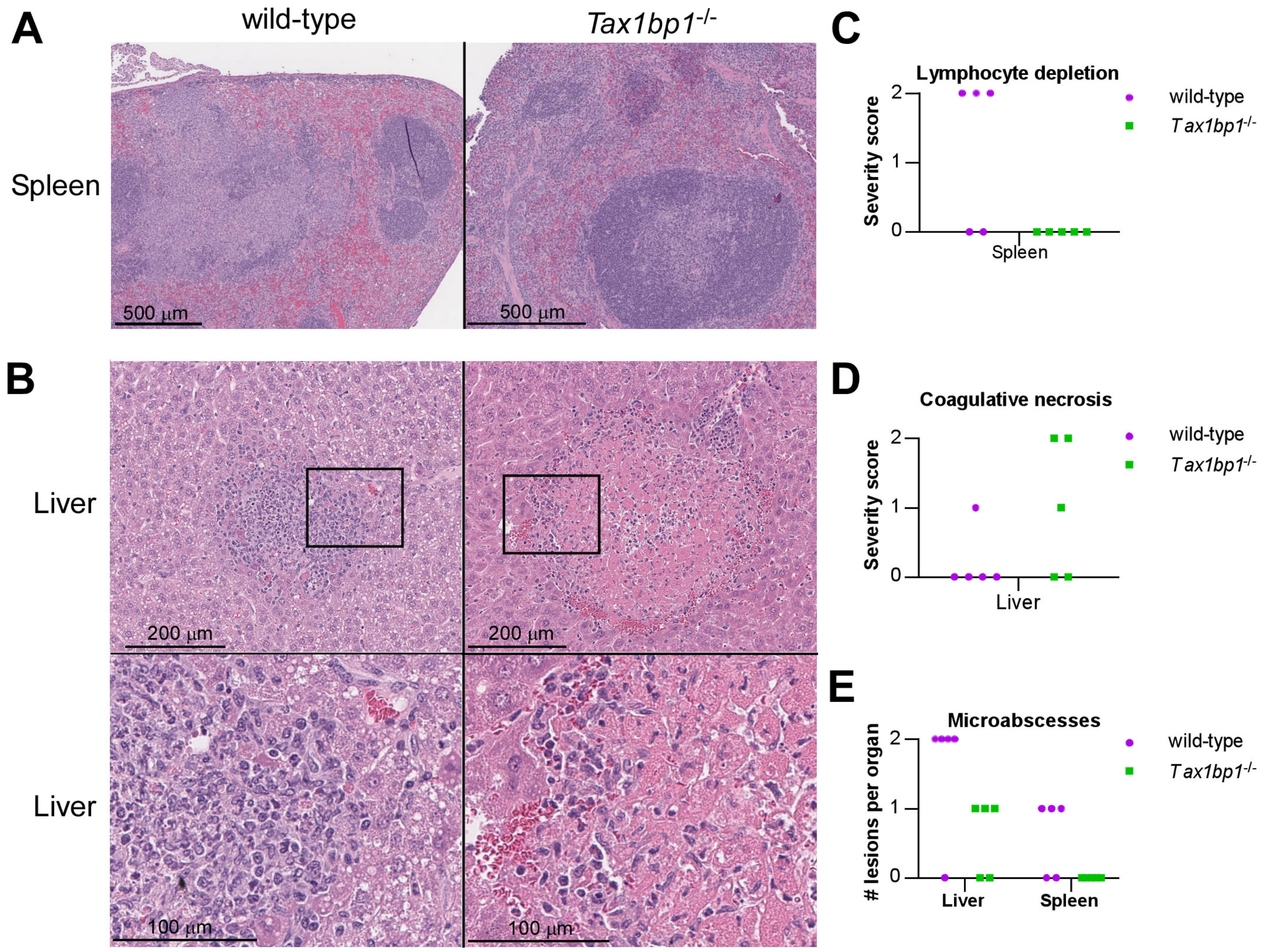

Figure 2-figure supplement 2

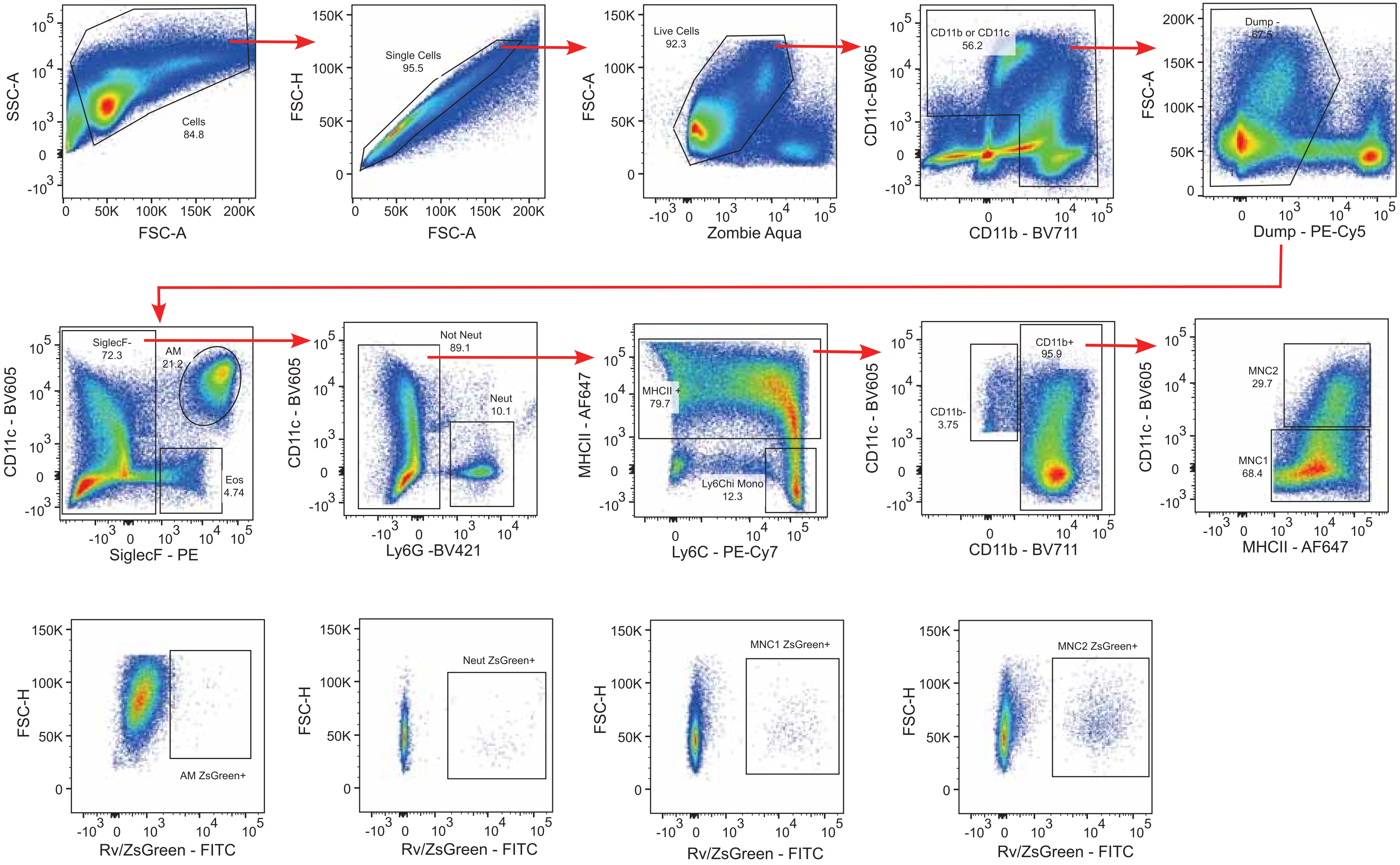

Figure 3-figure supplement 1

bioRxiv preprint doi: https://doi.org/10.1101/2024.12.16.628616; this version posted December 16, 2024. The copyright holder for this preprint (which was not certified by peer review) is the author/funder, who has granted bioRxiv a license to display the preprint in perpetuity. It is made available under aCC-BY 4.0 International license.

bioRxiv preprint doi: https://doi.org/10.1101/2024.12.16.628616; this version posted December 16, 2024. The copyright holder for this preprint (which was not certified by peer review) is the author/funder, who has granted bioRxiv a license to display the preprint in perpetuity. It is made available under aCC-BY 4.0 International license.

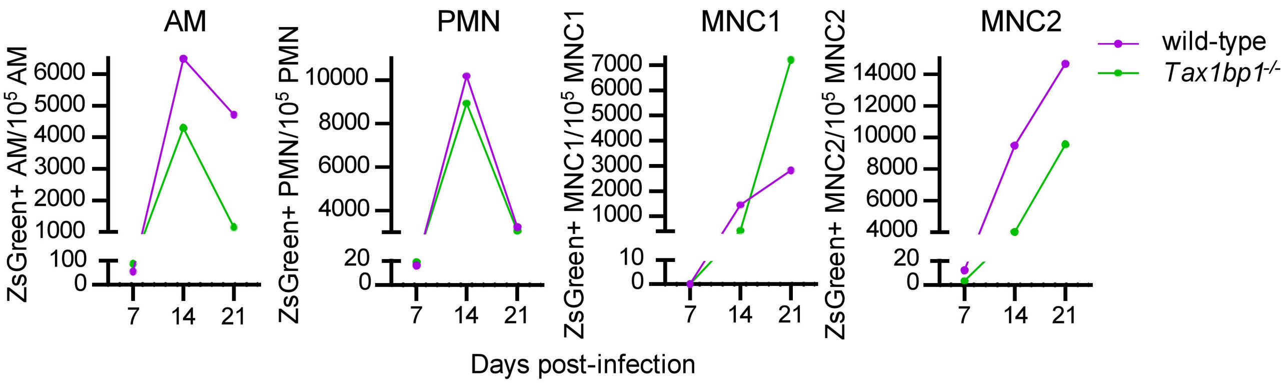

Figure 3-figure supplement 2

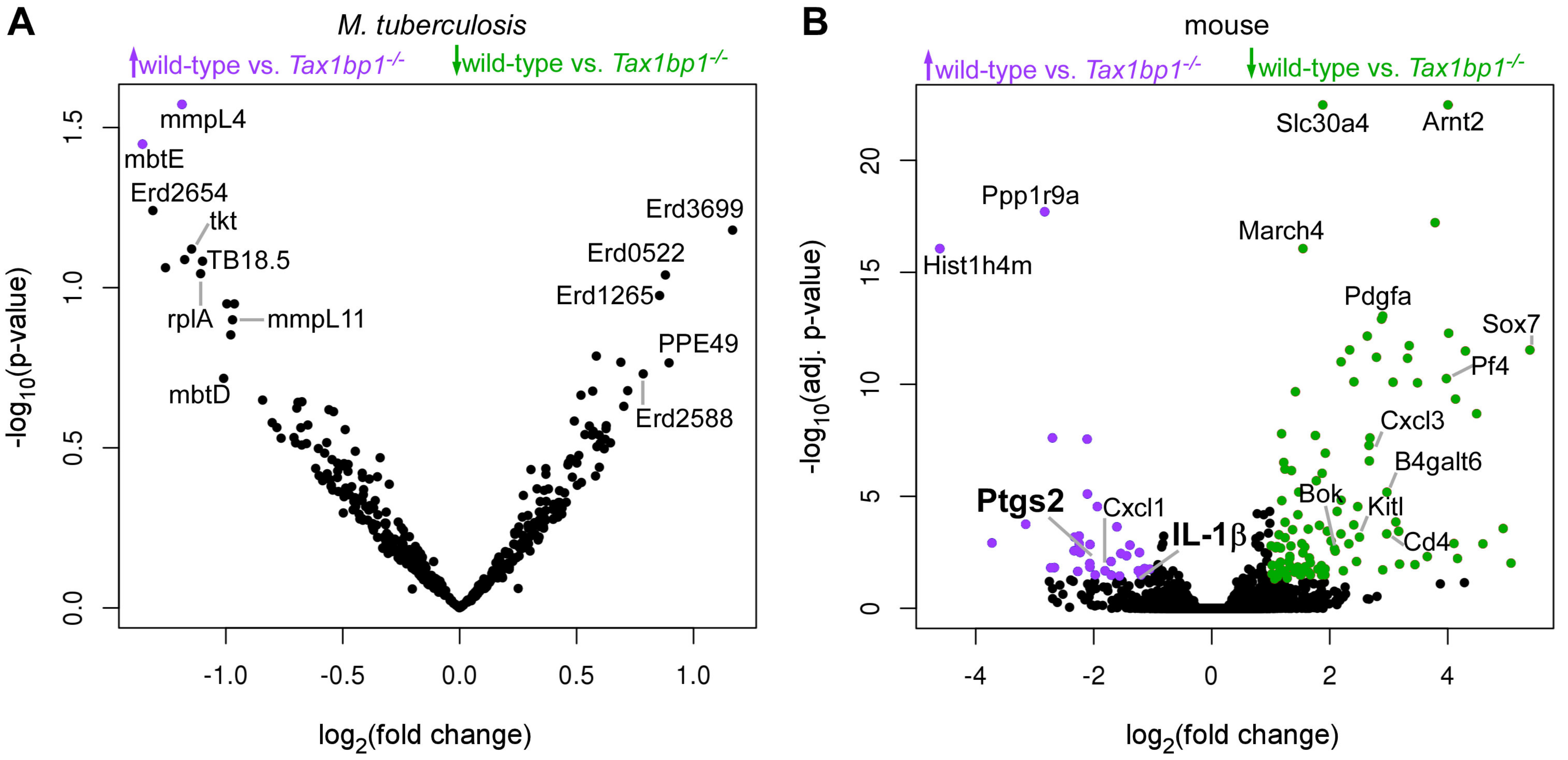

Figure 6-figure supplement 1

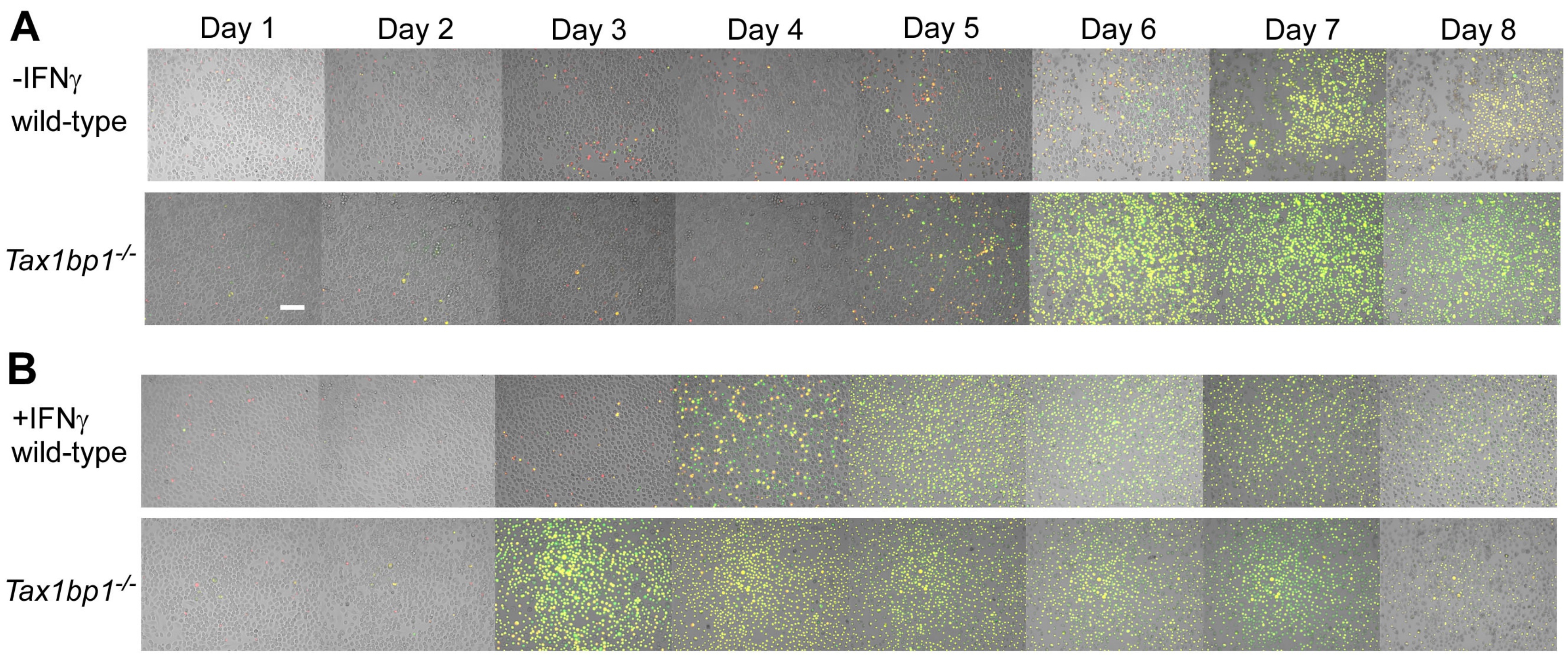

Figure 8-figure supplement 1

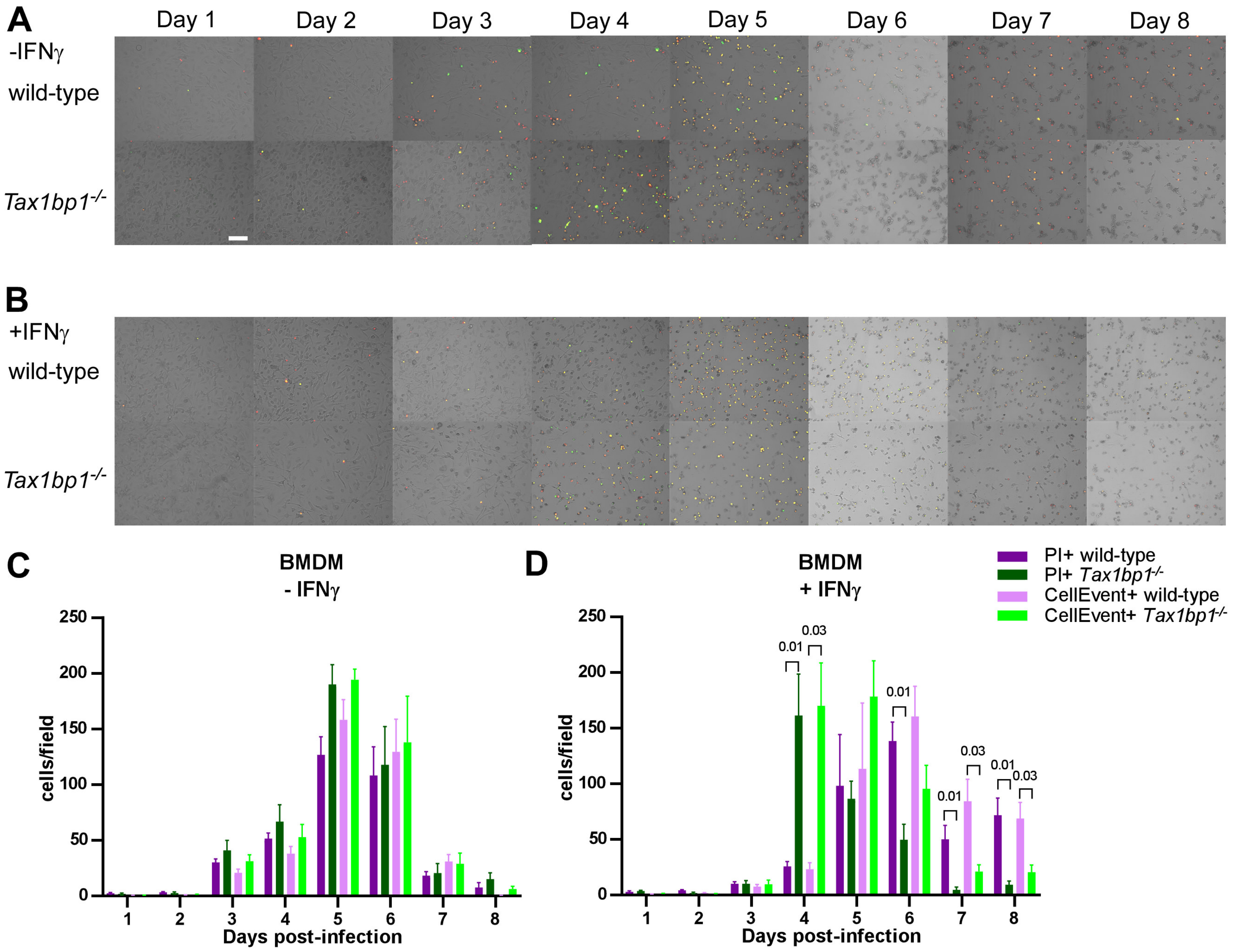

Figure 8-figure supplement 2

bioRxiv preprint doi: https://doi.org/10.1101/2024.12.16.628616; this version posted December 16, 2024. The copyright holder for this preprint (which was not certified by peer review) is the author/funder, who has granted bioRxiv a license to display the preprint in perpetuity. It is made available under aCC-BY 4.0 International license.

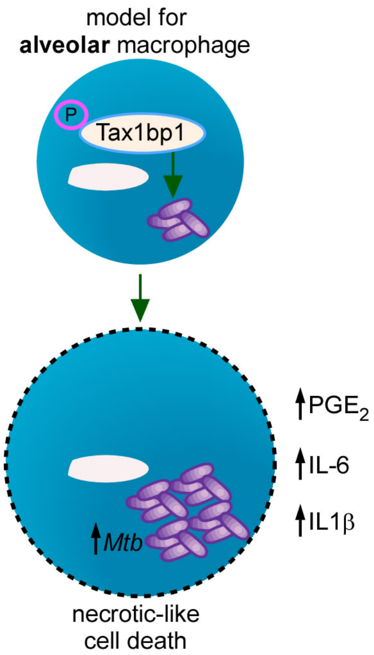

Figure 9-figure supplement 1
